# Adventures in Wonderland

**DOI:** 10.1371/journal.pgen.1005086

**Published:** 2015-04-16

**Authors:** Sarah Kucenas

**Affiliations:** University of Virginia, Charlottesville, Virginia, United States of America; Stanford University School of Medicine, UNITED STATES

For the last several decades, there has been little debate over the origins of central and peripheral glia. Central nervous system (CNS) glia, including oligodendrocytes, astrocytes, and radial glia, are specified from CNS neural precursors[1], whereas peripheral nervous system (PNS) glia, including Schwann cells and satellite glia, are derivatives of the neural crest [2]. However, this strict delineation of central versus peripheral glia is being challenged, and the implications could revolutionize human medicine. In the February issue of *PLOS Genetics*, Weider and colleagues report that a cell population with characteristics very similar to CNS oligodendrocytes can arise from satellite glia in the periphery with only the overexpression of a single transcription factor [3]. This work, in conjunction with several other recent papers [4–7], has us not only peering through Alice’s looking glass but also crossing straight through.

## Glial Surprises Down the Rabbit Hole

**Fig 1:**
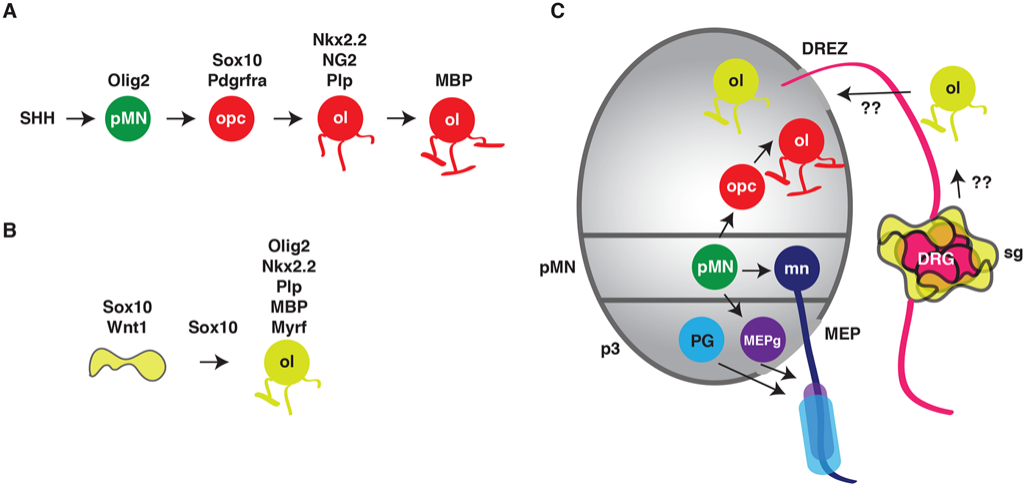
Conversion of satellite glia into oligodendrocyte-like cells. (A) In the ventral spinal cord, sonic hedgehog (SHH) induces the expression of Olig2 in pMN ventral spinal cord precursors (pMN, green circle). During gliogenesis, Olig2 initiates expression of Sox10 and other transcription factors, which leads to the specification of oligodendrocyte progenitor cells (opc, red circle). Differentiation of opcss results in mature, myelinating oligodendrocytes (ol). (B) Weider et al. present evidence that when overexpresed, Sox10 alone can convert a satellite glial cell (yellow cell) into an oligodendrocye-like cell (ol, yellow circle), which shares many of the same genetic signatures as CNS-derived oligodendrocytes. (C) Recently, a handful of papers have described that a subset of peripheral glia, including myelinating motor exit point glia (MEPg, purple cells) and perineurial glia (blue cells), arise from ventral spinal cord precursors. The work presented by Weider and colleagues raises the intriguing possibility that satellite glia (yellow cells) might normally give rise to oligodendrocyte-like cells and that they can migrate into the spinal cord via the dorsal root entry zone (DREZ) or that direct conversion of satellite glia into oligodendrocyte-like cells may be a valuable treatment option for diseases like MS if these cells could be induced to migrate directly into the spinal cord via the DREZ.

Decades of careful fate-mapping and lineage tracing has left us with the conclusion that the myelinating glia of the CNS, oligodendrocytes, are derived from spinal cord neural precursors [8]. The vast majority of these oligodendrocyte progenitor cells (OPC) originate from ventral spinal cord precursors found in the pMN domain. Their development is tightly regulated by a concert of morphogens and transcription factors, with sonic hedgehog (SHH) initiating the cascade and regulating the expression of oligodendrocyte transcription factor 2 (Olig2) [8]. Additional transcription factors come into play with Nkx2.2 and Sox10 acting as two players that, in coordination with Olig2, induce the expression of pro-myelinating genes, including proteolipid protein (Plp) and myelin basic protein (MBP) (Fig. 1A) [8]. In Multiple Sclerosis (MS), the immune system attacks and damages the myelin made by mature oligodendrocytes, ultimately leading to axon conduction deficits and severe disability. One promising avenue of research to combat this debilitating disease involves using transcription factor-mediated reprogramming to directly convert one cell type into another. In vitro, mouse fibroblasts were directly converted into OPCs using a cocktail of transcription factors that mirrors their development in vivo, including Sox10 and Olig2 [9,10]. Of these two transcription factors, Sox10 is often considered a master glial transcription factor as it is expressed by OPCs and all known peripheral glial lineages. However, these cell conversion strategies yield low efficiency of conversion and are rarely studied in vivo.

This paper by Weider et al. demonstrates that overexpression of Sox10 alone can convert an already Sox10-positive satellite glial cell within the dorsal root ganglia (DRG) into oligodendrocyte-like cells in vivo (Fig. 1B) [3]. This finding has three major implications: 1) Sox10 might be the only transcription factor required to convert cells into an oligodendrocyte-like lineage, 2) there is more than one way to make an oligodendrocyte, and 3) we need to learn more about these understudied satellite glia. The authors genetically dissect this glial conversion and find that, in contrast to OPC specification in the ventral spinal cord, where Olig2 initiates gliogenesis by promoting Sox10 expression, overexpression of Sox10 in satellite glia directly activates Olig2 and it is this step in cell conversion that is key to creating oligodendrocyte-like cells in the PNS (Fig. 1B). The authors then take this information to the next level and map a Sox10 evolutionarily conserved non-coding region (ECR) upstream of the *Olig2* transcriptional start site, which they call the Olig2 ECR (OLE). Weider et al. demonstrate via bioinformatics that the distal fragment of this OLE (OLEa) has fourteen potential Sox binding sites and this same OLEa is expressed in cells of the oligodendrocyte lineage in the spinal cord. Additionally, these oligodendrocyte-like cells in the DRG expressed Nkx2.2, Myrf, Plp1, and Mbp, but not Sox9, Pdgfra, and NG2, demonstrating that the conversion cascade in satellite glia is distinct from oligodendrogensis in the CNS (Fig. 1A and B). Intriguingly, when the authors attempt to recapitulate spinal cord genesis of oligodendrocytes in satellite glia by overexpressing Olig2, they observed none of the expected oligodendrocyte markers in the DRG. Therefore, conversion of satellite glia into oligodendrocyte-like cells is similar, but distinct from, normal spinal cord OPC specification.

The implications for this work are immense. In MS, repeated immune activation and attack of CNS myelin eventually leads to a loss of oligodendrocytes. Although attempted regeneration of this cell population is observed in many models of this disease, eventually, remyelination fails. These studies presented by Weider and colleagues describe, for the first time, the overexpression of a single transcription factor and its ability to convert satellite glia in the DRG into oligodendrocyte-like cells in vivo. This experimental design not only demonstrates the competency of satellite glia for conversion, but also reveals the possibility of using this method in human patients. The DRG sits immediately adjacent to the spinal cord and afferent sensory axons enter the CNS at the dorsal root entry zone (DREZ). If satellite glia can be converted to oligodendrocyte-like cells and induced to migrate into the spinal cord, these studies could represent a treatment option for patients with CNS myelinopathies, including MS, which avoid the ethical entanglements of stem cell biology and painful cell infusions.

## Through the Looking Glass

These fascinating studies not only impact human disease biology but also demand that we take a closer look at what we know about basic glial biology. What if satellite glia normally make oligodendrocytes? A second, much smaller population of spinal cord oligodendrocytes are derived from dorsal spinal cord precursors [11]. Could this population also have members that are derived from peripheral satellite glia that migrate into the spinal cord via the DREZ (Fig. 1C)? Recently, a handful of studies have investigated two peripheral glial lineages that arise from ventral spinal cord precursors and migrate into the periphery via the motor exit point (MEP) [4–6]. The studies in this paper in *PLOS Genetics* also raise the exciting possibility that satellite glia might normally contribute cells to the CNS. This would imply that it is not just axons that connect the CNS and PNS together into functional circuits but also glial populations that do this. Further investigation into the ultimate fate of these ectopic oligodendrocyte-like cells and their precursors, and close examination of whether these phenomenon occur under normal conditions, will yield intriguing insights into not only the basic biology of satellite glia but also how these cells may be useful in treating disease. And, not unlike Alice in Wonderland, we may have to follow glia through the looking glass (or spinal cord transition zones) to discover more about the nervous system and ourselves.

## References

[1] Rowitch DH. Glial specification in the vertebrate neural tube. Nat Rev Neurosci. 2004;5: 409–419. 15100723 10.1038/nrn1389

[2] Jessen K, Mirsky R. The origin and development of glial cells in peripheral nerves. Nat Rev Neurosci. 2005;6. 10.1038/nrn1746 16136171

[3] Weider M, Wegener A, Schmitt C, Küspert M, Hillgärtner S, Bösl MR, et al Elevated In Vivo Levels of a Single Transcription Factor Directly Convert Satellite Glia into Oligodendrocyte-like Cells. PLoS Genet. 2015;11: e1005008 10.1371/journal.pgen.1005008 25680202 PMC4334169

[4] Zawadzka M, Rivers L, Fancy S, Zhao C, Tripathi R, Jamen F, et al CNS-resident glial progenitor/stem cells produce Schwann cells as well as oligodendrocytes during repair of CNS demyelination. Cell stem cell. 2010;6. 10.1016/j.stem.2010.04.002 PMC3856868 20569695

[5] Kucenas S, Takada N, Park H-C, Woodruff E, Broadie K, Appel B. CNS-derived glia ensheath peripheral nerves and mediate motor root development. Nature neuroscience. 2008;11. 10.1038/nn2025 PMC2657597 18176560

[6] Smith CJ, Morris AD, Welsh TG, Kucenas S. Contact-mediated inhibition between oligodendrocyte progenitor cells and motor exit point glia establishes the spinal cord transition zone. PLoS biology. 2014;12. 10.1371/journal.pbio.1001961 PMC4181976 25268888

[7] Clark J, O’Keefe A, Mastracci T, Sussel L, Matise M, Kucenas S. Mammalian Nkx2.2(+) perineurial glia are essential for motor nerve development Developmental dynamics: an official publication of the American Association of Anatomists. 2014. 10.1002/dvdy.24158 PMC4180512 24979729

[8] Rowitch DH, Kriegstein AR. Developmental genetics of vertebrate glial-cell specification. Nature. 2010;468: 214–222. 10.1038/nature09611 21068830

[9] Najm FJ, Lager AM, Zaremba A, Wyatt K, Caprariello AV, Factor DC, et al Transcription factor-mediated reprogramming of fibroblasts to expandable, myelinogenic oligodendrocyte progenitor cells. Nat Biotechnol. 2013;31: 426–433. 10.1038/nbt.2561 23584611 PMC3678540

[10] Yang N, Zuchero JB, Ahlenius H, Marro S, Ng YH, Vierbuchen T, et al Generation of oligodendroglial cells by direct lineage conversion. Nat Biotechnol. 2013;31: 434–439. 10.1038/nbt.2564 23584610 PMC3677690

[11] Cai J, Qi Y, Hu X, Tan M, Liu Z, Zhang J, et al Generation of oligodendrocyte precursor cells from mouse dorsal spinal cord independent of Nkx6 regulation and Shh signaling. Neuron. 2005;45: 41–53. 15629701 10.1016/j.neuron.2004.12.028

